# Warm, lively, rough? Assessing agreement on aesthetic effects of artworks

**DOI:** 10.1371/journal.pone.0232083

**Published:** 2020-05-13

**Authors:** Eva Specker, Michael Forster, Hanna Brinkmann, Jane Boddy, Beatrice Immelmann, Jürgen Goller, Matthew Pelowski, Raphael Rosenberg, Helmut Leder

**Affiliations:** 1 Faculty of Psychology, Department of Cognition, Emotion, and Methods in Psychology, University of Vienna, Vienna, Austria; 2 Faculty of Historical and Cultural Studies, Department of Art History, University of Vienna, Vienna, Austria; 3 Vienna Cognitive Science Hub, University of Vienna, Vienna, Austria; 4 MECS, Leuphana University, Lüneburg, Germany; University of Hong Kong, HONG KONG

## Abstract

The idea that simple visual elements such as colors and lines have specific, universal associations—for example red being warm—appears rather intuitive. Such associations have formed a basis for the description of artworks since the 18^th^ century and are still fundamental to discourses on art today. Art historians might describe a painting where red is dominant as “warm,” “aggressive,” or “lively,” with the tacit assumption that beholders would universally associate the works’ certain key forms with specific qualities, or “aesthetic effects”. However, is this actually the case? Do we actually share similar responses to the same line or color? In this paper, we tested whether and to what extent this assumption of universality (sharing of perceived qualities) is justified. We employed—for the first time—abstract artworks as well as single elements (lines and colors) extracted from these artworks in an experiment in which participants rated the stimuli on 14 “aesthetic effect” scales derived from art literature and empirical aesthetics. To test the validity of the assumption of universality, we examined on which of the dimensions there was agreement, and investigated the influence of art expertise, comparing art historians with lay people. In one study and its replication, we found significantly lower agreement than expected. For the whole artworks, participants agreed on the effects of warm-cold, heavy-light, and happy-sad, but not on 11 other dimensions. Further, we found that the image type (artwork or its constituting elements) was a major factor influencing agreement; people agreed more on the whole artwork than on single elements. Art expertise did not play a significant role and agreement was especially low on dimensions usually of interest in empirical aesthetics (e.g., like-dislike). Our results challenge the practice of interpreting artworks based on their aesthetic effects, as these effects may not be as universal as previously thought.

## 1. Introduction

In Goethe’s seminal treatise about colors, first published in 1810, he wrote: “The colours on the plus side are yellow, red-yellow (orange), yellow-red (minium, cinnabar). The feelings they excite are quick, lively, aspiring” [1, p.306]. This statement was part of a chapter in which Goethe examined the effects of colors on perceivers, in which he also stated that “red-yellow gives an impression of warmth and gladness” [1, p.309]. Because of such effects, he considered color a central aspect of visual art: “colour considered as an element of art, may be made subservient to the highest aesthetical ends” [1, p.309].

Such attributes such as “warmth”, as typically discussed in the art historical or critical literature, are known as *aesthetic effects*, and are seen as specific responses in viewers to art, with theorists often assuming that they are caused by the specific nature of single elements in artworks, especially lines and colors [2]. This idea that simple visual elements such as colors (but also lines) elicit specific effects in humans, has been one of the foundations of modern discourses on art since the 18^th^ century and is still crucial for art critics and art historians today [2]: artworks with red tones, that is, “warm” colors, are described as “warm”. It is unclear, however, how much viewers indeed do share these effects of visual elements—how universal aesthetic effects in fact are. This is the aim of the present study, in which we test and quantify this notion of universality in aesthetic effects among both lays and art experts.

### 1.1. The relationship between aesthetic effects and cross-modal correspondences

Part of what is discussed as an aesthetic effect in art literature is reminiscent of what in modern psychology is known as *cross-modal correspondence*. Cross-modal correspondence refers to the input of two different sensory modalities being congruent, for example perceiving high-pitched versus low-pitched sounds to resemble bright versus dark colors [3,4]. Similarly, in the example of colors and temperature above, there might be a cross-modal correspondence of a tactile sensation of warmth with the visual input of “warm” colors such as red. Cross-modal correspondences, though not universal, seem to rely on a general basis: they can already be observed in very young children, suggesting a very basic learned or even innate trait [5,6] and can not only be found in humans but also in animals [7].

### 1.2. The claim for universality

The link between cross-modal correspondence and aesthetic theory is not surprising: historically, many theorists were inspired by the linking of different senses in a consistent way (as is the case in cross-modal correspondences). As such, theorists, when describing the link between colors and forms and their effects on humans, conceived these effects as objective (aesthetic) properties of the stimuli. These effects are often treated as essentially “perceptual” [8], that is, we can “see” that a line is dynamic in the same way as that we can see that it is curved. From a psychological perspective, one would rather speak of conceptual associations (i.e. an association between the concept “red” and the concept “warmth”), which lead to a certain aesthetic experience [9]. We mention this “perceptual” approach since it is the most common approach of theorists and itself led theorists to make an implicit assumption of universality.

Whether perceptual or conceptual, the assumption of universality in discussion of aesthetics is sometimes made explicit. Endell [10], for example, claimed that every color and every form cause the same feeling in all people. More often, it is an implicit aspect of theorists’ arguments—suggesting or implying that, of course it is obvious that a feature elicits a certain association or response. Nonetheless, when looking across the history of reports, things are not so clear. Different authors have proposed different aesthetic effects for the same color or type of line. Where red for Goethe [1], as well as for, for example, Edmund Burke [11], was a cheerful color, it was a brutal color for Franz Marc [12]. Such historical discrepancies cast first doubt on the universality of aesthetic effects, or raised the potential that not all aspects—colors, lines, curves—might evoke universal responses. Especially here, one might talk of lines, colors, and entire artworks, with perhaps changes in universality changing as a function of the complexity of a stimulus. This also raises the question of whether individual constituent parts—a curved versus straight line added to a painting—might always have the same additive impact on perception or associations in the whole—or not. Several theorists have also addressed the above discrepancies by raising the possibility that proper perception or perhaps making the “correct” associations requires a certain type of individual or training. Hume [13], for example, argued that a true judge has “a strong sense, united to delicate sentiment, improved by practice, perfected by comparison, and cleared of all prejudice”(p.17). Similarly, Sibley [14] argued that aesthetic perception “requires the exercise of taste, perceptiveness, or sensitivity” (p.421). Though it has been argued that (very) few people would meet or come close to meeting Hume’s standards [8,13,15] or perhaps those of Sibley, we feel that a true test of universality should at least attempt to take into account the notion that some observers could be more sensitive than others. We suppose that people who have a higher knowledge of art and interest in art might represent a more sensitive viewer. If these viewers are more sensitive, and there is a “true” effect, then they should agree more with each other on the aesthetic effects of an artwork than a group of less sensitive viewers. We address this in our study by comparing a group of psychology and art history students.

However, although there have been some attempts for empirical measurements of aesthetic effects (for an overview see [2]), the above issues are very much unresolved. We still lack a systematic test of the assumption of universality: it remains unclear, whether and to what extent aesthetic effects are individual or universally shared. That said, researchers in empirical aesthetics have sought universality in terms of preference for, or the relative beauty of, the golden section [16,17], fractals [18], rectangles [19], paintings [20], and across different aesthetic domains [21]. In addition, psychological studies have tested the universality of color associations [22], color-form associations [23,24], as well as associations with lines [25]. However, all of these latter studies have exclusively focused on single elements, without embedding those elements in complex, realistic stimuli such as artworks. It is thus not clear if they can inform us about the universality of the aesthetic effects of artworks. We address this in our study by using abstract artworks and single elements (lines and colors) extracted from these artworks.

### 1.3. The present study

**Fig 1:**
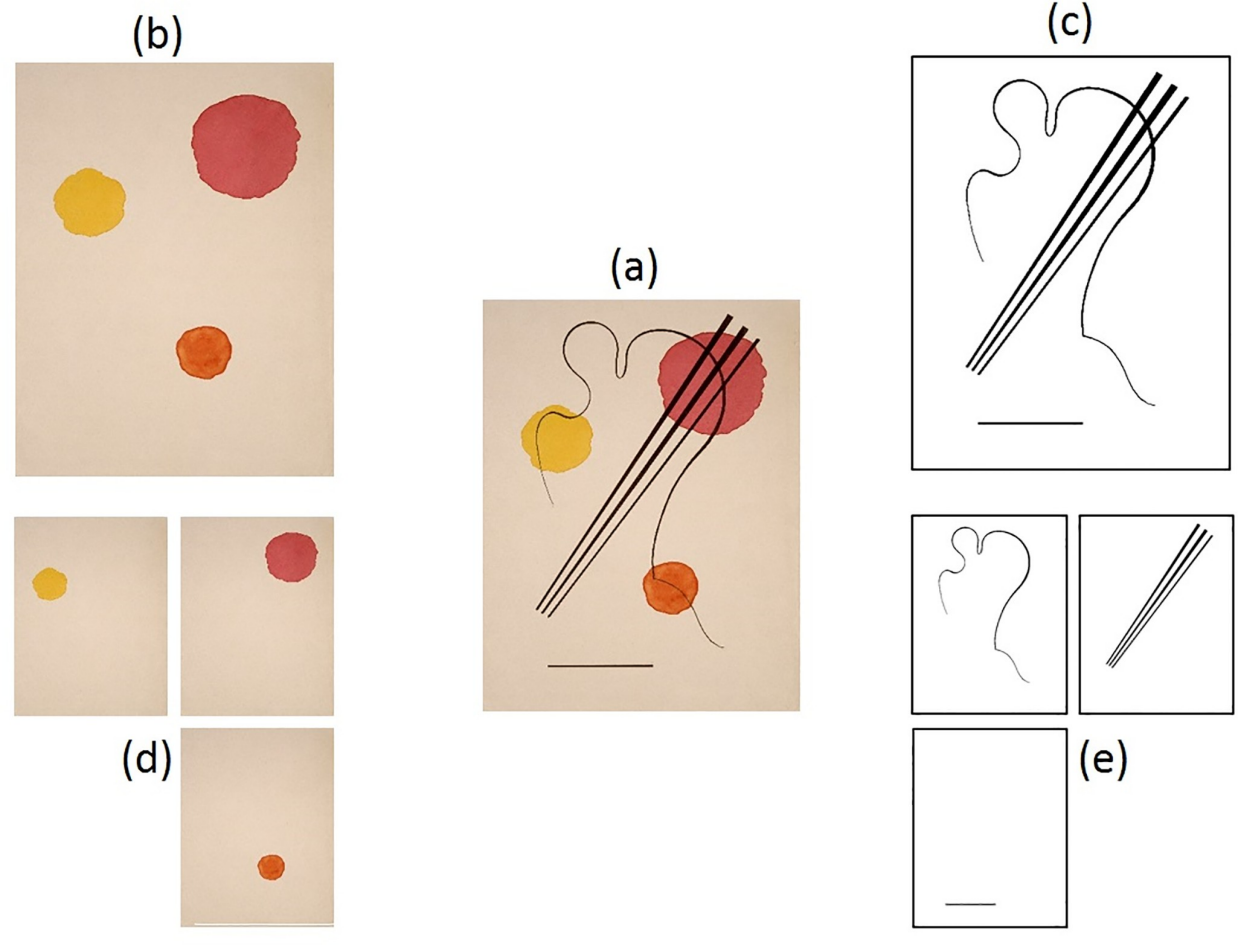
Separation from lines and colors from the artwork Wassily Kandinsky, Untitled, 1934, watercolor, ink, 31.6 × 24.6 cm, Paris, Centre Georges Pompidou. (A) Whole artwork, (B) the combination of colors, (C) combination of lines, (D) single colors and, (E) single lines.

The aim of this paper again is to test the notion of universality in aesthetic effects empirically, using both real artworks and their constituting elements. In order to trace back shared aesthetic effects, elicited by artworks, to possibly shared aesthetic effects, elicited by their constituting elements, we manipulated high-resolution reproductions of artworks digitally to separate single elements—lines a colors. This allowed us to investigate the single elements in isolation while also retaining the connection of these specific lines and colors, and the recorded ratings, to real artworks. We tested the aesthetic effects elicited by 1) the artwork as a whole, 2) the combination of all lines of the image, 3) the combination of all colors of the image, 4) the single lines of the image, and 5) the single colors of the image. We chose abstract artworks by Wassily Kandinsky, Fritz Winter, and Joan Mirò that could be divided into single components. As can be seen in Fig 1, we did not change the placement or size of the single elements. We measured aesthetic responses in 14 different dimensions, involving a representative choice of the 12 most common categories used in art literature (e.g., warm–cold, soft–hard, heavy-light), based on a previous historical review and empirical study [2]. In addition, we included the two most common dimensions in empirical aesthetics: dislike-like, uninteresting-interesting.

To measure the relative universality of evaluations made with the above scales, we relied on an index derived from generalizability theory [26,27]. Hönekopp [28] introduced this index as the beholder index (“bi”). Based on comparisons of variance components, this index allows quantifying the relative proportion of shared to individual variance in evaluations. Following its name, the beholder index reflects how much of the variance is due to private evaluation. For example, if a bi score is .75, 75 percent of the variance in an evaluation of a stimulus is private evaluation and the remaining 25% is due to shared evaluations across the sample of participants. Therefore, to support the notion that aesthetic effects are universally shared, we would need to find low beholder indices where less than half of the variance is due to private evaluation, meaning that more than half of the variance is due to shared evaluations. This measure is often used in studies assessing agreement on facial attractiveness (see e.g. [29] which also offers a comparison with other statistical measures of agreement). To our knowledge the method has only been used in relation to artworks twice [20,30]. Both of these studies assessed agreement of artworks in comparison to natural stimuli and found that agreement on artworks was lower than agreement on natural stimuli.

In a second step, we investigated the potential role of knowledge about art and interest in art. Finally, we conducted a replication study to assess the robustness of our results.

## 2. Method

The bi method estimates variance components that can be interpreted as the observed variance attributed either to the participant or the stimulus (see [20], p.2 for a comprehensive explanation of the estimation of the variance components). In order to do this, participants need to rate each stimulus twice. The repeated measure allows for estimation not just of how much participants agree with each other on a rating (shared evaluation) but also how much participants agree with themselves on the repeated rating (private evaluation). These variance components can then be combined into the beholder index (“bi”). Hönekopp [28] provided two different beholder indices: bi_1_ and bi_2_. Both use the variation explained by the interaction between the participant and the image as an indicator of how much variation is attributable to private evaluation. The crucial difference is that bi_2_ additionally uses the variation explained by the participant as an additional indicator for private evaluation. There are two ways to interpret this additional variation: first, one could argue that the differences between participants in overall ratings independent from specific stimuli are not meaningful but simply reflect differences in scale use and not differences in evaluation (bi_1_ is indicated). Second, one could argue that these differences reflect a meaningful part of private evaluation (bi_2_ is indicated). We have no strong opinion on which of the two indices is preferable, but rather think that the appropriate value probably lies in between both indices. We chose to use bi_1_ in the main body of text and in all the figures. However, to ensure completeness, we include bi_1_ and bi_2_ in the tables of results.

### 2.1. Design

In order to calculate beholder indices we measured each participant twice. Therefore, participants completed two sessions of one hour each with a minimum of two days in between. We used a blocked design for each session, with participants first completing Block 1, followed by an unrelated filler task, and then Block 2. Block 1 was always a rating task of aesthetic effects for the single lines and colors with a total of 28 stimuli (see Fig 1, Panels d and e). Block 2 was always first a rating task of aesthetic effects for the combination of lines and colors (6 stimuli), and second the same task for the three artworks as a whole (see Fig 1, Panels a, b, and c). The filler task was included to help with concentration and to avoid fatigue. We chose this design in order exclude the influence of seeing the whole artwork on the rating of the individual elements. We anticipated that if participants saw the whole artwork before (or in between) rating the individual elements, the whole artwork could potentially influence their rating of the single elements to a large extent. This design leaves open the possibility for the reverse effect, namely that the rating of the single elements influences the whole artwork. However, we saw this as less problematic for two reasons. First and foremost, theoretically one would assume that the individual elements contribute to the overall impression of an image constructed of these elements. That is, one assumes that lower-level features inform the impression of the whole image as evidenced by research on lower-level features such as curvature [31–34] or symmetry [35,36]. Thus, it would be impossible to exclude this influence experimentally. Additionally, since the single elements are presented randomly and not blocked per artwork it is unlikely that participants would keep track of which elements belong “together”, while at the same time remembering each rating they gave for approximately 40 minutes (Block 1 took about 30 minutes, followed by a 10-minute filler task).

### 2.2. Participants

We collected data of a total sample of 107 participants. Seven participants were excluded due to dropping out between the sessions, data loss, or low variance in the answers. Thus, the final sample consisted of 100 participants (24 men, age range: 19–48 years, *M* = 25.12, *SD* = 5.56) with groups of 50 art history students (9 men, age range: 19–48 years, *M* = *M* = 24.44, *SD* = 4.73) and 50 psychology students (15 men, age range: 19–43 years, *M* = 25.81, *SD* = 6.26). All participants received a compensation of €30 for their participation at the end of the second session. Psychology students were recruited through the online system of the faculty of psychology of the University of Vienna. The system offered a short description of the study and participants could sign up for the study and pick the timeslots in which they wanted to participate. Art history students were recruited in art history lectures at the same university. They received a verbal explanation of the study and could then give their email address if they were interested in participating. They were afterwards contacted by email to make an appointment. Participants were required to speak German fluently. Data collection took place from 3^rd^ of May 2017 to 29^th^ of June 2017 in the psychology lab. The study was carried out in accordance with the Declaration of Helsinki and the was approved by the ethical committee of the University of Vienna.

### 2.3. Materials

#### 2.3.1. Aesthetic effects

To measure aesthetic effects, we used 14 rating scales. Each rating scale was a 7-point Likert-type bipolar scale (also referred to as a semantic differential), meaning that each aesthetic effect was represented by opposite pairs as poles of the scale, for example “warm–cold”. Based on [2] we used the terms: negative–positive, passive–active, lively–still, happy–sad, aggressive–peaceful, soft–hard, warm–cold, heavy–light, smooth–rough, bodily–spiritual, masculine–feminine, intrusive–cautious. We added the most common categories of empirical aesthetics: dislike-like, and uninteresting-interesting. In each case the left term was represented by 1 and the right term by 7. Left term and right term are here used to refer to the opposite sides of the dimension such as “warm-cold”, in this case warm would be referred to by 1 and cold by 7. The specific wording of questions for each scale was as follows, “This image appears… warm/cold” (In German: “Dieses Bild wirkt…warm/kalt”). For liking the question was rephrased to “I like/I do not like this image” (in German: “Dieses Bild…Gefällt mir/Gefällt mir nicht”) in order to stay grammatically correct (please note that in German the grammar between liking and the other scales is much more similar than in English).

#### 2.3.2. Stimuli

We used high-quality reproductions of the following artworks: Wassily Kandinsky, *Untitled*, 1934, watercolor, ink, 31.6 × 24.6 cm, Paris, Centre Georges Pompidou; Joan Miró, *Untitled*, 1961, color etching and aquatint, 31.7 × 24 cm (cat. raisonné no. 292); Fritz Winter, *Siebdruck 6*, 1950, screen print, 50 × 65 cm. For each image, we digitally extracted the single elements as illustrated in Fig 1, resulting in a total set of 37 stimuli (3 whole artworks; 3 combination of colors, 3 combination of lines, 11 single colors, and 17 single lines). We selected relatively homogenous artworks, given that the agreement between observers has been shown to vary as a function of the heterogeneity of stimuli used [28]. All “lines” in the images were black lines; this was done in order to not have our lower-level categories of “lines” and “colors” be confounded. In addition, all colors were circle-shaped with the exception of one element in the Winter image that had a bow-like shape.

#### 2.3.3. Filler task

In Session 1, the filler task was the Vienna Art Interest and Art Knowledge questionnaire (VAIAK) [37]. This provided a filler task as well as a manipulation check for the assumed higher art interest and art knowledge of the art history students. In Session 2, the filler task was an unrelated experimental task.

### 2.4. Procedure

Upon arrival in the lab for the first session, participants received and signed an informed consent form. They were instructed by the experimenter to follow the instructions on the screen and complete the task (Block 1). After completing Block 1, the experimenter started the filler task for the participant. When the participant finished the filler task, the experimenter started Block 2 for the participant, where again, they were instructed to follow the instruction on the screen and complete the task. Within the blocks, participants saw an image and rated that image for all aesthetic effects before moving to the next image. The question order of the aesthetic effects was randomized, as was the image presentation order.

Upon arrival in the lab for the second session, participants went through the same procedure, the only exception being not signing the informed consent again. After the second session was finished, participants were thanked, debriefed, and received their compensation. Single sessions normally lasted between 40 and 60 minutes, depending on the participant.

## 3. Results

In a first analysis, we checked whether the two groups indeed differed on art interest and knowledge. Art history students were more interested in art (M = 64.90, SD = 7.58) and had more knowledge about art (M = 15.46, SD = 4.39) than lay people (M_interest_ = 39.82, SD = 12.58; M_knowledge_ = 5.24, SD = 3.19, as assessed by two independent samples t-tests, ps < .001). This indicated that our selection of art history students and psychology students was a suitable way to select two groups that meaningfully differed on these variables.

To assess the proportion of private versus shared evaluation, we next calculated the bi. Once again, as a main metric, this can be interpreted for where it lies either above or below .50 (50% variance due to either shared or private taste). Note, as mentioned by Leder et al. [20], methods to calculate standard errors and confidence intervals for variance components exist [38], however, because variance components are usually not normally distributed “summarizing the precision of a variance component estimate by giving an approximate standard error is woefully inadequate” (p.19) [39]. We therefore do not report standard errors or confidence intervals. Unfortunately, this makes it impossible to test for significance, either between the different image types or between the different groups of participants. Therefore, all comparisons here are purely descriptive, using the above general metric, and should be interpreted as such.

**Fig 2:**
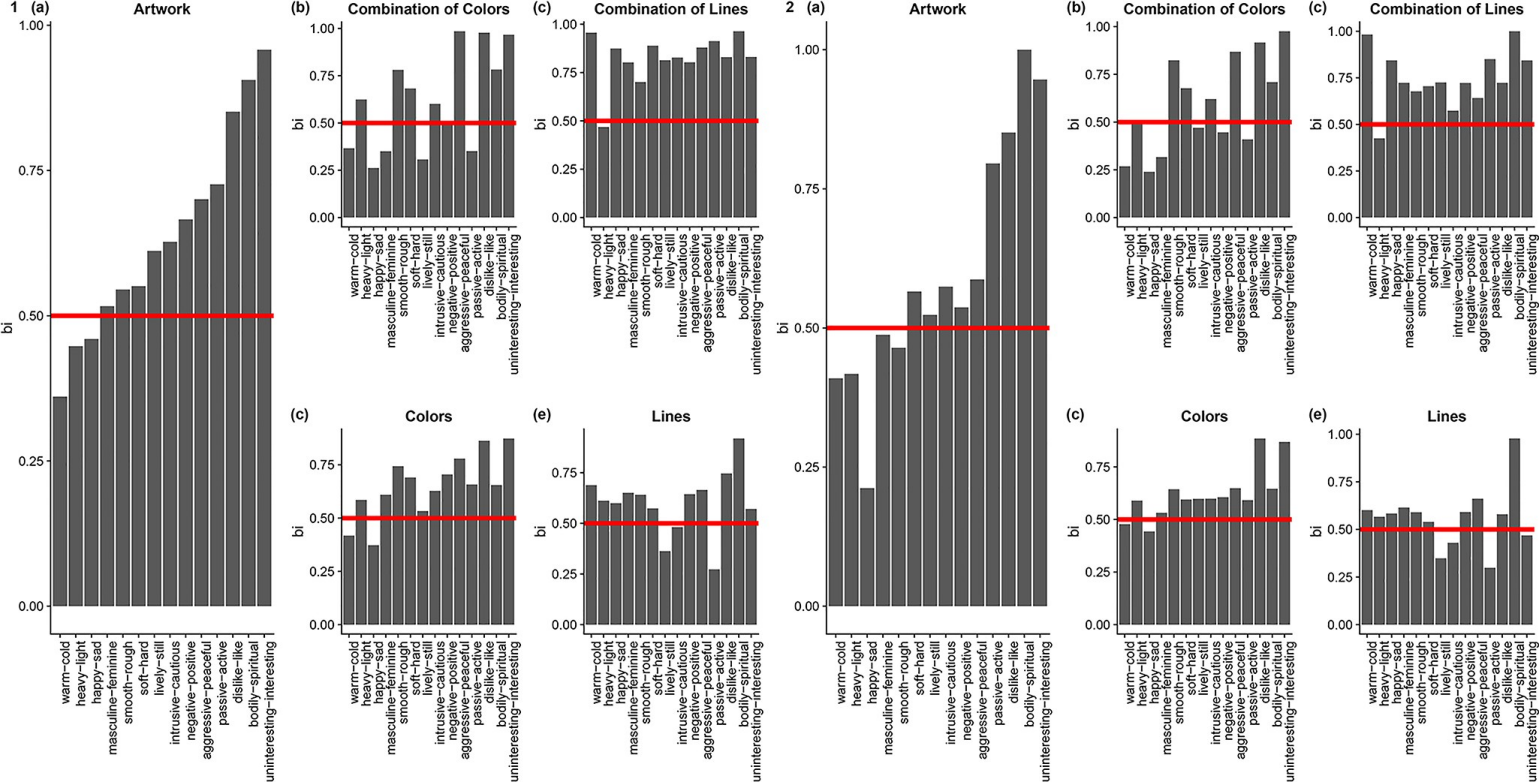
Histogram of the beholder index as separated by experts and type: (A) artwork, (B) combination of colors, (C) combination of lines, (D) single colors, and (E) single lines. The left-side panel (Panel 1) shows the data from the original study (N = 100) and the right-side panel (Panel 2) shows the data from the replication study (N = 50).

First, we calculated beholder indices of the aesthetic effects over our total sample, separately for each image type as reported in Table 1 and illustrated in Fig 2 (Panel 1). This showed that across image types there was consistently a higher level of private evaluation for the majority of terms. Interestingly, image type seems to play a major role in determining the agreement amongst raters. For the whole artwork, the aesthetic effects of warm, heavy, and happy were mainly determined by shared evaluation.

**Table 1:** Beholder indices over the total sample of the original study separated by image type.

Terms	Artworks		Combination of Lines		Combination of Colors		Lines		Colors	
	bi_1_	bi_2_	bi_1_	bi_2_	bi_1_	bi_2_	bi_1_	bi_2_	bi_1_	bi_2_
Warm–cold	**.34**	**.36**	.96	.98	**.33**	**.34**	.68	.82	**.42**	.52
Heavy–light	**.43**	.52	**.46**	.55	.55	.63	.61	.70	.58	.75
Happy–sad	**.41**	**.47**	.88	.93	**.20**	**.28**	.60	.67	**.36**	.53
Masculine–feminine	.50	.50	.79	.79	**.32**	**.39**	.64	.72	.60	.70
Smooth–rough	.53	.57	.71	.75	.79	.83	.64	.73	.74	.84
Soft–hard	.54	.63	.89	.90	.67	.69	.57	.65	.69	.81
Lively–still	.63	.71	.83	.87	**.31**	**.45**	**.36**	**.43**	.53	.74
Intrusive–cautious	.64	.67	.84	.91	.60	.74	**.48**	.60	.63	.82
Negative–positive	.64	.64	.81	.81	**.43**	.50	.64	.68	.70	.81
Aggressive–peaceful	.67	.73	.88	.89	.96	.97	.67	.71	.77	.88
Passive–active	.75	.86	.92	.95	**.35**	**.42**	**.26**	**.34**	.65	.82
Dislike–like	.86	.86	.84	.85	.96	.96	.73	.79	.86	.93
Bodily–spiritual	.92	.94	.96	.98	.74	.85	.92	.94	.65	.84
Uninteresting–interesting	.97	.97	.84	.87	.94	.95	.54	.62	.86	.92

Bold values are values that meet our self-defined cut-off of < .50.

This appeared to indicate that aesthetic effects are not universally shared but rather are highly determined by private evaluation. In addition, there are large differences between the different image types, essentially making agreement dependent on image type. It is worth noting that, in general, people agreed more on the aesthetic effects of the artwork (3 out of 14 effects that are mainly determined by shared evaluation) than on the aesthetic effect of the elements of those artworks (with the exception of the combination of colors). Furthermore, the two variables that researchers in empirical aesthetics are mostly interested in, liking and interest, are highly determined by private evaluation. This is in line with previous findings [20,21,30]. In sum, what this seems to indicate is that we did not find support for the assumption of universality of aesthetic effects.

**Fig 3:**
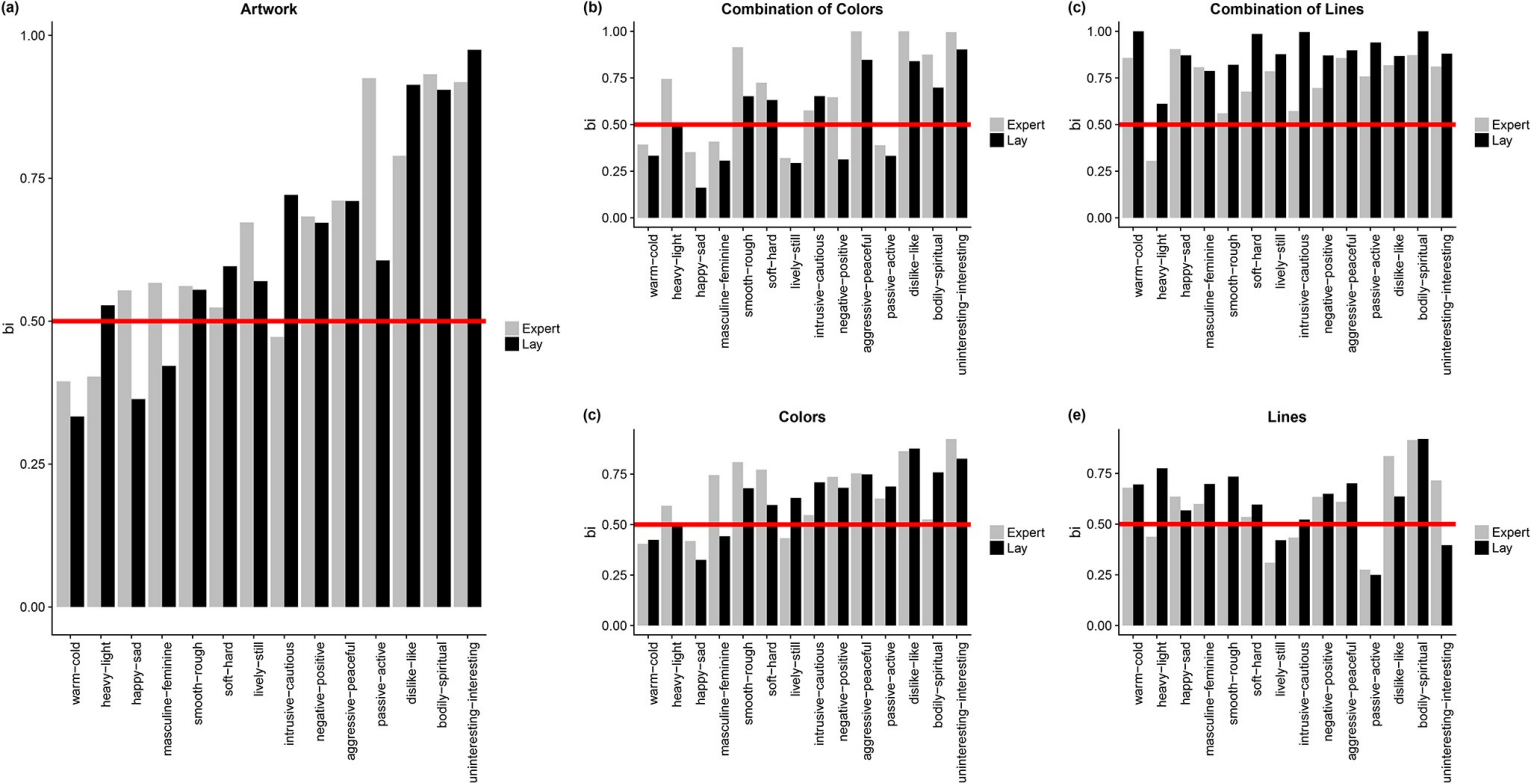
Histogram of the beholder index as separated by experts (light gray) and lays (dark gray) and image type: (A) artwork, (B) combination of colors, (C) combination of lines, (D) single colors, and (E) single lines.

In a second step, we calculated beholder indices of the aesthetic effects separately for our two groups (art history students and lay people) and for each image type as reported in Table 2 and illustrated in Fig 3. This showed that experts do not consistently agree more than lay people (in which case the dark grey bar of experts should be consistently lower than the light grey bar). The only exception was the combination of lines where experts consistently agreed more on the aesthetic effects than lay people. As mentioned, there is no way to test for significant differences in the beholder indices.

**Table 2:** Beholder indices over the total sample of the original study separated by image type and expertise.

Terms	Expertise	Artworks		Combination of Lines		Combination of Colors		Lines		Colors	
		bi_1_	bi_2_	bi_1_	bi_2_	bi_1_	bi_2_	bi_1_	bi_2_	bi_1_	bi_2_
Warm–cold	Lay	**.31**	**.35**	.99	.99	**.32**	**.33**	.69	.81	**.41**	.51
	Expert	**.37**	**.39**	.86	.97	**.35**	**.35**	.67	.83	**.41**	.52
Heavy–light	Lay	.51	.54	.60	.63	.50	.56	.77	.82	.50	.74
	Expert	**.38**	.51	**.28**	**.47**	.62	.71	**.44**	.57	.59	.72
Happy–sad	Lay	**.36**	**.46**	.86	.87	**.15**	**.27**	.56	.63	**.31**	.55
	Expert	**.46**	**.49**	.93	.98	**.26**	**.30**	.64	.71	**.41**	.53
Masculine–feminine	Lay	**.41**	**.43**	.77	.77	**.30**	**.36**	.69	.73	**.43**	.61
	Expert	.54	.54	.79	.80	**.36**	**.43**	.59	.71	.74	.79
Smooth–rough	Lay	.55	.55	.80	.80	.67	.73	.73	.78	.67	.80
	Expert	.54	.61	.61	.72	.92	.94	.50	.65	.81	.88
Soft–hard	Lay	.58	.66	.98	.98	.62	.62	.59	.70	.59	.77
	Expert	.51	.60	.68	.79	.69	.77	.54	.60	.77	.84
Lively–still	Lay	.57	.67	.88	.89	**.28**	**.44**	**.41**	.51	.62	.79
	Expert	.70	.76	.79	.84	**.34**	**.47**	**.31**	**.32**	**.43**	.68
Intrusive–cautious	Lay	.70	.70	.99	.99	.64	.80	.51	.64	.70	.88
	Expert	.52	.68	.59	.82	.59	.69	**.44**	.56	.55	.73
Negative–positive	Lay	.68	.68	.87	.87	**.31**	**.41**	.64	.65	.68	.80
	Expert	.63	.63	.69	.76	.54	.58	.63	.70	.73	.83
Aggressive–peaceful	Lay	.70	.71	.90	.90	.86	.90	.70	.73	.74	.87
	Expert	.60	.76	.81	.86	.99	.99	.62	.69	.75	.87
Passive–active	Lay	.61	.76	.95	.96	**.32**	**.39**	**.23**	**.36**	.68	.85
	Expert	.94	.97	.70	.77	**.41**	**.46**	**.27**	**.29**	.62	.80
Dislike–like	Lay	.91	.92	.86	.86	.85	.87	.63	.69	.87	.93
	Expert	.81	.82	.84	.86	.99	.99	.81	.86	.85	.94
Bodily–spiritual	Lay	.91	.94	.97	.99	.71	.79	.92	.93	.75	.88
	Expert	.94	.96	.89	.94	.77	.91	.91	.94	.53	.81
Uninteresting–interesting	Lay	.98	.98	.88	.88	.90	.91	.38	.48	.82	.91
	Expert	.95	.97	.83	.87	.98	.99	.68	.73	.90	.94

Bold values are values that meet our self-defined cut-off of < .50.

In sum, what this seems to indicate is that there is no empirical support for the notion that art experts would consistently agree more on the aesthetic effects due to their shared knowledge. As outlined above, the absence of an adequate method to calculate standard errors and confidence intervals for variance components makes it hard to assess the precision of the point-estimates of the bis. We therefore carried out a replication study in order to address the replicability of the findings.

## 4. Replication

For the replication study, we only included lay participants because we did not find an effect of expertise. Our sample consisted of 50 lay participants (17 men, age range: 18–38 years, *M* = 21.98, *SD* = 4.23). Methods and analysis were identical to the original study. The findings are reported in Table 3 and illustrated in Fig 2 right-side panel (Panel B). A comparison between Panel A and Panel B of Fig 1 reveals that we replicated the main findings of our original study. However, even though the general pattern of findings was similar, it is also apparent that the point estimate of the bi can shift. It therefore seems inadvisable to interpret the point estimate of the bi in and of its own. A better way is to interpret the tendency whether the rating is mainly determined by private or mainly by shared evaluation.

**Table 3:** Beholder indices over the total sample of the replication study separated by image type.

Terms	Artworks		Combination of Lines		Combination of Colors		Lines		Colors	
	bi_1_	bi_2_	bi_1_	bi_2_	bi_1_	bi_2_	bi_1_	bi_2_	bi_1_	bi_2_
Warm–cold	**.41**	.52	.98	.99	**.27**	**.27**	.60	.77	**.48**	**.48**
Heavy–light	**.42**	.54	**.43**	**.48**	.50	.63	.57	.68	.59	.66
Happy–sad	**.21**	**.40**	.84	.85	**.24**	**.24**	.58	.67	**.44**	.51
Masculine–feminine	**.49**	.56	.72	.74	**.32**	**.33**	.62	.66	.53	.61
Smooth–rough	**.46**	.63	.68	.70	.82	.87	.59	.69	.64	.78
Soft–hard	.56	.64	.71	.71	.67	.69	.54	.64	.60	.74
Lively–still	.52	.59	.73	.81	**.47**	.52	**.35**	**.46**	.60	.80
Intrusive–cautious	.57	.63	.57	.60	.62	.75	**.43**	.50	.60	.71
Negative–positive	.54	.63	.72	.73	**.45**	**.49**	.59	.66	.61	.63
Aggressive–peaceful	.59	.61	.64	.64	.87	.95	.66	.73	.65	.71
Passive–active	.80	.83	.85	.88	**.41**	.51	**.30**	**.39**	.59	.77
Dislike–like	.85	.87	.72	.74	.92	.92	.58	.65	.89	.92
Bodily–spiritual	.99	.99	.99	.99	.71	.84	.99	.99	.65	.81
Uninteresting–interesting	.95	.95	.84	.85	.98	.98	.47	.60	.87	.91

Bold values are values that meet our self-defined cut-off of < .50.

## 5. Discussion

This study is—to the best of our knowledge—the first attempt to test the universality of aesthetic effects by using original material (high-quality reproductions of artworks) and rating scales based on the terms most common in art discourses. The results suggest that in general we cannot assume universality of aesthetic effects. Across image types, most terms were not agreed upon and there was no consistent pattern in which terms people agreed on. This rebuttal of universality challenges a fundamental assumption on which both historical and contemporary discourses on art are based.

The suggestion of low agreement in ratings of aesthetic effects is reinforced by the finding that we did not see more agreement among experts. The individual differences in knowledge about art or in interest in art do not matter for the degree of agreement in the perception of aesthetic effects: if people have more knowledge or interest, they do not agree more and have no higher level of shared evaluation than people who have less knowledge or interest.

However, it has to be noted that when taking into account the whole artwork we did find consistent agreement on the aesthetic effects of warm–cold, happy–sad, and heavy–light. Thus, our findings do not suggest that there is no universality at all; rather, they imply that the agreement is definitely not as strong as often assumed. In addition, universality does not need to imply perfectly shared evaluation. For example, we know that people have quite some shared evaluation of faces, however previous research has shown that even for faces the bi lies between .40 [20] and .34 [30]. It would therefore be too strong to conclude that there is no agreement at all; nonetheless, we can conclude that the agreement is lower than presupposed.

Analyzing the details of the results shows that, whereas the amount of agreement is generally low, it does vary according to the type of images and aesthetic dimension. First, there seems to be more agreement when rating complex abstract artworks as a whole, rather than their single elements. This would argue against the assumption that the effect of the whole results from the sum of its parts—the elementary effects of single lines and colors—and rather supports a Gestalt approach where the sum is considered more than the sum of its parts. Second, as already mentioned, three (warm–cold, happy–sad, and heavy–light) out of twelve scales used to measure aesthetic effects did show high agreement scores—both in the initial study and in its replication. Third, there is less universality in taste (liking and interest) than in rating aesthetic effects (the 12 other scales we used). This third result questions the search for general laws of aesthetics which lies at the heart of numerous studies on empirical aesthetics, while the first two findings question aesthetic theories from several centuries. We will now discuss this in more detail.

First, given the literature about aesthetic effects and cross-modal correspondence, the finding that there is more agreement on the complex image than on the single elements is surprising. A reason for this may be that, although the colors and lines in our study varied, they did not have a systematic variation: if one wanted to test for a cross-modal correspondence between a visual (e.g. colors, lines) and haptic domain (the haptic domain is implemented in our design by the aesthetic effects of smooth–rough, soft–hard, warm–cold), one would normally focus on one aspect of colors (e.g. brightness). However, in our design the different color stimuli did not only vary in brightness but also in hue and saturation. Similarly, the lines differed in orientation, thickness, etc. Seen this way, even the lower-level features in our design had several aspects to them. This variation may have obscured some of the cross-modal correspondences. That said, when we investigate these effects in an aesthetic context we are dealing with stimulus material that will always have relatively high variation. When comparing one image to another it is unlikely that we find them only differing in, for example, line thickness. That is not to say that it is not worthwhile to investigate how line thickness contributes to our aesthetic perception, but only that if these cross-modal correspondences only occur at a very low-level and do not translate to complex images such as artworks (because there are simply too many other factors influencing our judgement), the theory that cross-modal correspondences would be applicable to such complex stimuli is seriously challenged. To test the theory that these effects apply to artworks one has to test it with real artworks as stimuli and this is exactly what we did in this study. Though our results do not indicate that these effects are not applicable, they do indicate that people do not agree on these effects. That is, the correspondences are highly idiosyncratic.

In general, this finding suggests that results obtained using stimuli material of low-level features may not generalize to complex artworks. Therefore, we would recommend future research to assess the effect of interest on the level of interest, meaning that if the theory proposes an effect on the level of the artwork the study should use artworks as stimuli material. If images of lower level features (e.g. symmetrical patterns) are used instead this may limit the external validity of the study.

These interpretations are strengthened by the finding that this general pattern was replicable. However, the results of the replication also highlight the imprecision of the point estimates of the bi. It seems that the point estimate in and of itself is not a suitable point of reference. Rather, it can be used to describe a central tendency. Thus, one can say from the bi that participant’s ratings are either mainly based on private evaluation or mainly based on shared evaluation. In the case that they are mainly based on shared evaluation, we postulate that this means that people agree on these effects.

Second, why do people agree on warm–cold, happy–sad, and heavy–light but not on the other aesthetic effects? There have been some studies showing a relation between color (or one of its attributes, i.e. hue, brightness, and saturation) and perceived warmth or weight [40–43]. Research has also shown a connection between brightness of colors and positivity [22,44–48], which could speculatively contribute to explaining the happy–sad finding. However, we also included a negative–positive scale as such. This scale only showed agreement in the combination of colors and not in any of the other image types. Therefore, these studies may, perhaps, explain the findings somewhat, but their explanatory power is limited due to the fact that they focused only on single elements (namely colors) rather than considering complex images.

Furthermore, we took a systematic approach in our study, testing all scales of aesthetic effects for all stimuli. However, the practice of any art critic and or art historian is different: they carefully select some terms for some pictures and would rather not use many other terms. We therefore assume, and intend to test in a follow-up study, that when using scales judged as appropriate to specific stimuli, the amount of agreement might be significantly higher: warm–cold, happy–sad, and heavy–light seem to be appropriate for the kind of abstract paintings used in this study. Warm-cold and happy-sad are apparently appropriate for colors (single colors and combination of colors), but not for lines. Whereas, lively-still and active-passive are appropriate for single lines.

This study of course also comes with other caveats and demands for future research. First, on the methodological level, we did not include a measure of emotional experience either before, after, or during our study. Since it is generally accepted that art can evoke emotions [9,49,50], and additionally that our emotional states can influence our aesthetic experience [51,52], this can be a confounding factor in our design.

Second, our sample included more females than males (out of our total sample of 150 participants only 41 were male). This is not surprising, since the majority of students of psychology and art history (from which we drew our samples from) are female. Although there is no reason to assume that there would be a gender difference, this nonetheless could be a confounding factor and also limits generalization (see below).

A third limitation is that our expert sample was based solely on art history students. This limits our conclusions that expertise does not make a difference. A sample of artists or others (e.g. graphic designers, illustrators, etc.) engaged in creating artworks and thus working more actively and directly with artistic elements, may be a more appropriate comparison group, or at least a comparison group that is also appropriate and may lead to different results. In an ideal case, we would have included non-student expert samples (there is also a case to be made for including art-professionals such as art critics, for example), however, this is unfortunately hard to realize especially when aiming for relatively large sample sizes since these people are harder to recruit.

Fourth, apart from our comparison between experts and non-experts, we had a relatively homogenous sample: all of our participants were what has been termed “WEIRD” (Western Educated Industrialized Rich Democratic) [53], in addition to the above mentioned large proportion of females. This makes it unclear to what extent our results are generalizable to other populations. That said, given that we found rather low agreement in this homogeneous sample it seems unlikely to find more agreement in a more heterogeneous sample. Furthermore, though it is theoretically possible that other samples may show higher agreement this seems rather implausible, given the fact that the theories we tested originate from Western art history and were thus tested in a culturally congruent context.

In conclusion, despite the limitations, our findings challenge a fundamental assumption on which both historical and contemporary discourses on art are based. Future work on aesthetic effects as well as other art-related research relying on the assumption of universality should assess how likely this assumption is to hold in their specific case and adjust accordingly. We should be careful to not “throw away the baby with the bathwater” and declare all theory void based on one empirical study, but at the same time these finding should be taken seriously by theorists. We hope that our empirical work inspires future theoretical work and creates a discourse between the humanities and the sciences, because only then our understanding of aesthetics can increase.
